# To Remove Powder Stains

**Published:** 1901-07

**Authors:** 


### Abstracts and Selections.

### To Remove Powder Stains.

Dr. C. R. Clark reports a case of severe powder burn of the face in which he succeeded in removing all the staining resulting from the imbedded grains in less than two days by the application of three parts of hydrogen dioxide and one part of glycerine. He gives the credit for this treatment to Dr. J. Neely Rhoades, of Philadelphia, who several weeks ago by the use of undiluted hydrogen dioxide successfully removed powder stains from the face of an Italian woman in whose case other methods of treatment had entirely failed of success.—*American Medicine.*

